# Food preference and gender are associated with medial/frontopolar prefrontal regions functional near-infrared spectroscopy responses during eating: An exploratory study in young adults

**DOI:** 10.1371/journal.pone.0343481

**Published:** 2026-08-03

**Authors:** Yusuke Takatsuru, Yuka Sekine, Hideyasu Sato, Tomoko Osera

**Affiliations:** 1 Division of Multidimensional Clinical Medicine, Department of Nutrition and Health Sciences, Toyo University, Asaka, Saitama, Japan; 2 Division of Applied Nutrition, Department of Nutrition and Health Sciences, Toyo University, Asaka, Saitama, Japan; 3 Department of Food Life Sciences, Toyo University, Asaka, Saitama, Japan; Niigata University, JAPAN

## Abstract

Even if they have no dementia, some elderly people find it difficult to imagine the food they may want to eat. However, research on appetite assessment in older adults with poor appetite remains limited, due to the lack of a method that can easily measure brain function in clinical settings. In this study, we aim to clarify the relationship between activity in the food-dependent medial/frontopolar prefrontal regions (MFPR) and food preferences and consumption frequency in healthy young adults, with the aim of establishing methods for assessing food preferences and appetite in elderly individuals with poor appetite using functional near-infrared spectroscopy (fNIRS). All young participants were asked about their food preferences and intake frequency using a questionnaire, and they were instructed to look and then eat the control dish (CD; typical Japanese home-cooked meal) and their preferred dish (PD; each participant purchased the dish themselves on the day of the experiments) on separate days, and the activity of the MFPR in each participant was recorded by fNIRS. We found that the activity of the MFPR during “eating” were differ depend on the dishes, from 9 min after the start eating. Moreover, we found that the preference of foods was also associated with the activity of MFPR during “eating”. These exploratory findings suggest that MFPR fNIRS signals during eating may be associated with food preference-related variables.

## Introduction

With the increasing number of elderly people in Japan (the percentage of the elderly has increased to 29.1%) [1], we are facing the problem of appetite loss among them. Chewing problems, depressive symptoms, and polypharmacy among others decrease the appetite of the elderly [2–5]. Even if they have no dementia, some elderly people find it difficult to imagine the food they may want to eat. In the case of the elderly with swallowing problem, they “don’t want to eat” because they can only eat food paste. Morley and Silver [6] categorized the appetite loss in the elderly into; decreased demand, decreased hedonic qualities, decreased feeding drive, and increased activity of satiety factors. Appetite loss aggravates malnutrition disorders such as frailty in the elderly [7,8]. However, studies of appetite loss in the elderly are still insufficient [4]. One reason for this may be that research into the role of the brain in appetite and related food choices by easily measuring brain function in clinical settings has not progressed.

Brain activities during eating have been assessed using neuroimaging techniques such as functional magnetic resonance imaging (fMRI), positron emission tomography, and magnetoencephalography [9]. These techniques can accurately show the localization of brain activities in response to taste stimulation. However, they require their head to be hold completely stationary, which is far from the usual condition during eating. From this perspective, functional near-infrared spectroscopy (fNIRS) is one of the noninvasive techniques for detecting brain activities similarly to fMRI. Although fNIRS has a poorer spatial resolution than fMRI [10], it has a higher temporal resolution than fMRI, and a subject can move more freely and perform a wider range of tasks such as eating [11,12]. As previously reported, the activity of MPFC area 10 during the task of eating food, such as preferred and nonpreferred taste, has been studied well by fNIRS [9]. Moreover, the fNIRS setup is easy to move around compared with the MRI setup and thus, we can record signals even if participants cannot come to a special area.

We eat provided food (in our own homes, in hospitals, or schools; thus, we cannot choose) or purchased food (we can choose what we want eat from shops or restaurants). What happens when we can choose the food and decide what to eat? The information of vision, olfaction, and taste affects eating behavior [13]. Such information is first processed at visual cortex, olfactory cortex, and frontal operculum/insula. Then, the amygdala, orbitofrontal cortex, and pregenual cingulate cortex process the information as a reward/affective value for food/eating. These brain areas also moderated by the lateral prefrontal cortex and hunger-related neurons. Finaly, area 10 of the medial prefrontal cortex (MPFC; decision-making), the cingulate cortex (for action output), the striatum (for habit), and the lateral hypothalamus (for autonomic and endocrine responses) control the eating behavior and related actions. Eating behavior is, thus, very complicated and affected by several factors.

Aim of this study is to clarify the relationship between activity in the food-dependent medial/frontopolar prefrontal regions (MFPR) and food preferences and consumption frequency in healthy young adults, with the aim of establishing methods for future assessment in older adults with poor appetite using fNIRS. The activity of MFPR be recorded by fNIRS even if the participants cannot speak or write theirs answers to questionnaires. Thus, if we can find some correlation between the activity of MFPR which recorded by fNIRS, and questionnaire responses for categorizing the participants, we may provide exploratory information for the elderly people especially for those having trouble in saying/writing what they want to eat. For this purpose, we prepared two types of dishes. The control dish (CD; typical Japanese home-cooked meal) provided in institutions such as hospitals) and their preferred dish (PD; each participant purchased the dish themselves on the day of the experiments). We examined whether fNIRS responses during looking at and eating food were associated with food preference, intake frequency, and food-related knowledge. These factors could associate with the food-related preferences, behaviors, and knowledge of eating. Some of them are correlated with different brain regions [9] and are difficult to observe by fNIRS. However, if we find some relationship of these factors with MFPR activity using fNIRS, it may be useful for clarifying the appetite of the elderly. Because this is a pilot study to establish the methods before applying them to elderly participants, this study was performed on young participants.

## Materials and methods

### Procedure

The study was approved by the ethics committee of Toyo University (TU2020–011-TU2021-H-009, TU2020–011-TU2021-H-009-TU2021-H-023-TU2023-H-021, TU2020–011-TU2021-H-099-TU2021-H-023-TU2023-K-046) and performed in accordance with the Declaration of Helsinki. The experiments were conducted on participants who applied themselves through oral or posted recruitment from 31/01/2022–16/07/2025. After the experiments were completed, one subject self-reported a history of eating disorders and was therefore excluded from the study. All 68 participants (33 males, 21.6 ± 0.2 years old; 35 females, 21.2 ± 0.2 years old) provided written informed consent before the experiments. In this study, menstrual cycles were not recorded in order to reduce the psychological burden on female participants. Three female participants were left-handed and the rest were right-handed. To gather methodological knowledge, we observe healthy young participants in this study. After obtaining informed consent, all participants completed questionnaires on food intake frequency and preference and underwent the food intake test by fNIRS recording.

### Measures

#### Food intake frequency and preference.

All participants were asked about their food intake frequency, food preferences, food-related behavior, and knowledge of food using a questionnaire before starting the food intake test (S1 Table and S2 Table). Their knowledge of seasonal foods was analyzed on the basis of the score of summation (one correct answer was given one point with 20 points as the maximum). Food intake frequency was determined using the Frequency Questionnaire Based on Food Groups (FFQg) (Ver. 6, Addin software of EIYO-KUN Ver. 9, Kenpakusya, Bunkyo-ku, Tokyo, Japan) [14,15] which included the information on gender and age (we only obtained the birth year. The participants were defined to have been born on January 1st, and their age was determined on the basis of the date given in the FFQg form).

#### Food intake test.

All participants ate the control dish (CD; typical Japanese home-cooked meal) and their preferred dish (PD; each participant purchased the dish themselves on the day of the experiments) on separate days. All experiments were performed during lunchtime (11:00–14:00, JST). The target amount of CD was set at 34% of 1600 kcal of the daily intake for elderly women because of all the subjects can finish eating. The macronutrient balance for protein, fat, and carbohydrate (PFC) energy production was in accordance with the Dietary Reference Intakes (DRI) for Japanese [16]. CD included nine components (grains, fish, pork, soy, sugar, vegetables, sea weed, and seafood), whose adequacy was assessed in accordance with DRI (see also S1 Text). The participants purchased PD themselves under the following conditions: (1) can be eaten in the university including drinks (easy to carry, highly perishable, without alcohol), (2) at amounts that can be consumed completely, and (3) the cost of food was covered by research funds. After eating the dishes, some of the participants evaluated the dishes using the visual analog scale (VAS. 0–100 mm). VAS ratings were collected from a subset of participants for descriptive purposes and were not included in the present analyses. The participants received a compensation of 3,000–5,000 Japanese yen (which included travel fee and not excluded the cost of purchasing PD). The CD and PD conditions were not matched for food type, nutritional composition, portion size, appearance, temperature, or sensory properties.

### fNIRS recording

The activity of MFPR during eating was recorded using a multichannel fNIRS unit operating at wavelengths of 770 and 840 nm (OEG-16H; Spectratech Inc., Yokohama) to measure temporal changes in the concentrations of oxygenated hemoglobin (oxyHb), deoxygenated hemoglobin (deoxyHb), and total hemoglobin (totalHb) as described previously [17]. S1 Fig. shows the position of eight infrared irradiation probes and eight infrared reception probes for the calculation of signals from the brain for information in sixteen channels. The probes were located 30 mm apart. The positions of the probes were based on the international 10–20 system used in electroencephalography and the center of the probe pad was located on Fpz (i.e., the midpoint between Fp1 and Fp2). The recording interval was 82 ms, and ten data points were averaged for smoothing, obtaining data every 0.82 s [9]. We used autocalibration in the OEG16H device before recording and if the gain was between 2000 and 100, we obtained the channel for the data analysis. If the gain was beyond this range, we reattached the probes. After reattaching and the signal gain was is detected more than our standard (more than 3 signal from cHs 7–10), we proceeded with the experiments and the data that were obtained beyond the range was omitted in the analysis. We removed the component of body movement by using the formula below [18]:


(ΔHbOFΔHbDF)=1kF−kS(−kS1−kFkSkF)(ΔHbOΔHbD),

where ∆HbO_F_ is the amount of signal change for oxyhemoglobin associated with cerebral function, ∆HbD_F_ is the amount of signal change for deoxyhemoglobin associated with cerebral function, ∆HbO is the amount of signal change for oxyhemoglobin detected by fNIRS, and ∆HbD is the amount of signal change for deoxyhemoglobin detected by fNIRS. k_F_ and k_S_ are coefficients. We used 0.6 for k_F_ [18] and k_S_ was calculated for each participant using the algorithm in OEG-16H. Although this method of removing motion components may be insufficient, it is a standard feature of the device and can be easily used, which is an advantage when considering future clinical applications.

The signal data of each probe were calculated by the device using the formulas below;


$$\begin{array}{c} oxyHb(mMmm)={\text{1}\text{0}}^{\text{4}}\times \frac{ed\text{2}\times \left[-{Log}_{\text{1}\text{0}}\left(\frac{V\text{1}}{V\text{1}\text{0}}\right)\right]-ed\text{1}\times [-{Log}_{\text{1}\text{0}}\left(\frac{V\text{2}}{V\text{2}\text{0}}\right)]}{ed\text{2}\times eo\text{1}-ed\text{1}\times eo\text{2}}, \end{array}$$

$$\begin{array}{c} deoxyHb(mMmm)={\text{1}\text{0}}^{\text{4}}\times \frac{eo\text{2}\times \left[-{Log}_{\text{1}\text{0}}\left(\frac{V\text{1}}{V\text{1}\text{0}}\right)\right]-eo\text{1}\times [-{Log}_{\text{1}\text{0}}\left(\frac{V\text{2}}{V\text{2}\text{0}}\right)]}{eo\text{2}\times ed\text{1}-eo\text{1}\times ed\text{2}}, \end{array}$$

where eo1 is the molar absorption coefficient of oxyHb by 840 nm (cm^-1^/M), ed1 is the molar absorption coefficient of deoxyHb by 840 nm (cm^-1^/M), eo2 is the molar absorption coefficient of oxyHb by 770 nm (cm^-1^/M), ed2 is the molar absorption coefficient of deoxyHb by 770 nm (cm^-1^/M), V1 is the current signal evoked by 840 nm, V10 is the basal signal evoked by 840 nm, V2 is the current signal evoked by 770 nm, and V20 is the basal signal evoked by 770 nm. In our methods, eo1 (= 1022), ed1 (= 692.36), eo2 (= 650), and ed2 (= 1311.88) were automatically determined. For filtering, we use low-pass fast Fourier transform and the baseline was corrected by linear fit in device.

**Fig 1:**
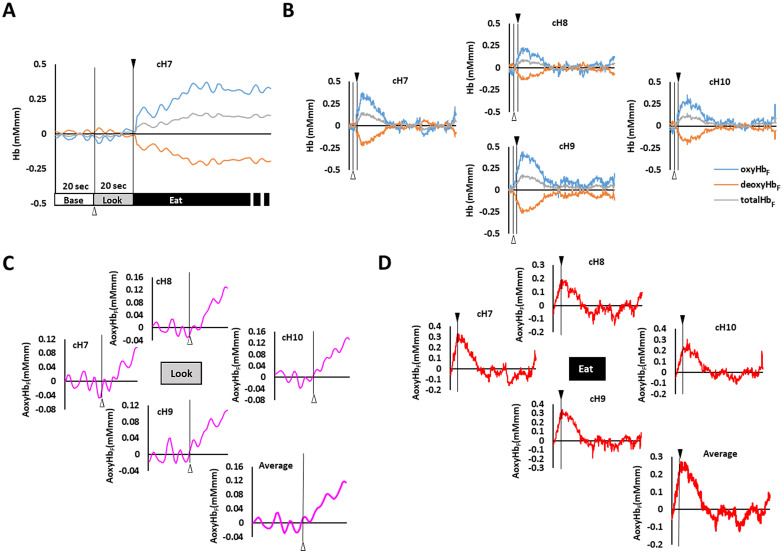
Typical signals from the MFPR (A, B) and calculated oxyHbF (C, D) in participants during CD eating.

For each fNIRS recording, 20 s was allocated for the baseline (participants were instructed to relax and wait), 20 s for “just looking”, and a sufficient time for “eating” (the first 10 min was used for analysis to exclude response changes due to fullness and/or increasing the blood sugar concentration). The data on oxyHb_F_ from each channel were averaged every 0.82 s, and the average signal (average oxyHb_F_; AoxyHb_F_ was defined as the mean oxyHb_F_ during each condition minus the mean oxyHb_F_ during the baseline period) of cHs 7–10 was used as the signal from MPFC [19–24]. However, due to the relatively low spatial resolution of fNIRS and probe placement, the measured signals may reflect broader prefrontal activity, including the frontopolar cortex (BA10), rather than specific MPFC subregions. In addition, fNIRS cannot adequately measure deeper cortical regions such as the ventromedial prefrontal cortex. Thus, we estimated the signals from cHs 7–10 as, medial/frontopolar prefrontal regions (MFPR) response. Then, we observed the “just-looking” condition (19.66 s) and “eating” condition (599.65 s) (Fig 1).

Signals from each channel were calculated as described in Methods, and oxyHb_F_ (normalized to the baseline recording) is presented. In each experiment, the food (CD or PD) was presented to participants after 20 s of baseline recording. After another 20 s of just looking, they started eating. Recording continued until they finished eating, and the first 10 min of recording was used for analysis.

### Statistical analyses

To investigate the dynamic changes in MFPR responses during the “eating” condition, the total duration was divided into five equal 2-min blocks. A two-way repeated measures analysis of variance (ANOVA) was performed with Condition (CD vs. PD) and Time (five blocks) as within-subject factors using IBM SPSS Statistics (version 31, IBM Corp., USA). Greenhouse-Geisser correction was applied to adjust for violations of sphericity where appropriate. For post-hoc analysis, pairwise comparisons with Bonferroni corrections were used to specify the time-window blocks in which significant differences between the conditions occurred.

Parts of the questionnaire were analyzed by principal component analysis (PCA) using Excel analysis (BellCurve, Japan). The validity of the factor selection used in PCA was determined using the Kaiser-Meyer-Olkin measure, and the anti-image correlation measure of sampling adequacy (reduce the factor if the anti-image correlation matrix is lower than 0.5) and Bartlett’s test using IBM SPSS Statistics (version 31, IBM Corp., USA). We also performed the multiple regression analysis using Excel analysis (BellCurve, Japan). The average (19.66 s for “just-looking” and five blocks of 119.60 s for “eating”) of AoxyHb_F_ were used in the analysis. As a sensitivity check, linear mixed-effects models with participant as a random intercept yielded fixed-effect estimates similar to those of the reported models. Therefore, the final analyses were reported using multiple regression models with dish condition included as a fixed-effect dummy variable (CD = 0, PD = 1) (N_obs = 136, N_participants = 68). Additionally, binary logistic regression analysis was performed after participants were divided into two groups according to whether they had any dislike foods. Although four variables exhibited potential associations in the univariable analysis (p < 0.1), the final multivariable binary logistic regression model included only the two variables with p < 0.05. This restriction was applied to adhere to the rule of thumb of at least 10 events per variable, given the smaller group size (no disliked foods, n = 22), thereby preventing overfitting of the model. Differences were considered significant at *p* < 0.05 or *α* < 0.05. D*a*ta are shown average ± standard error of the mean (SEM).

## Results

### The activity of MFPR during “eating” the food is influenced by the food within the 9–10 min time-window block

**Fig 2:**
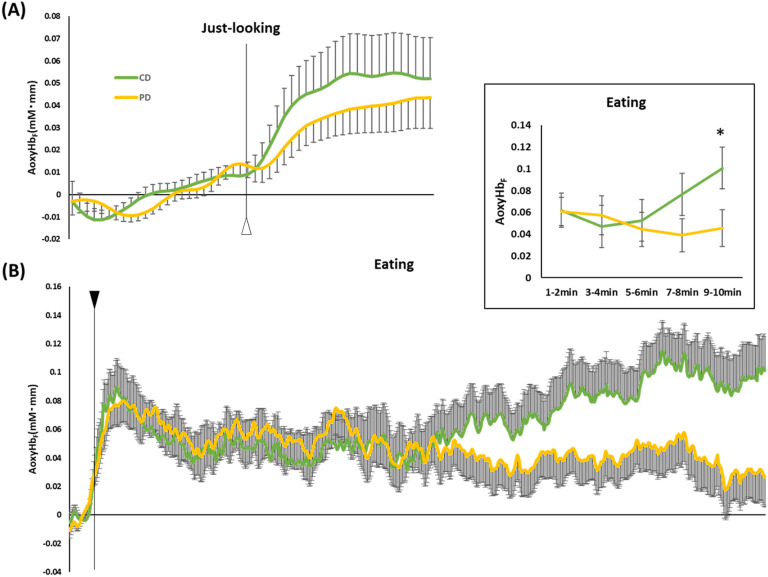
Average trace of AoxyHb_F_ under the Just-looking (A) and Eating (B) conditions.

As shown in Fig 2, the value of AoxyHb_F_ during “just-looking” did not differ significantly between the CD and PD conditions. In contrast, AosyHb_F_ during “eating” differed between the CD and PD conditions, particularly in the late part of the experiment. The two-way repeated measures ANOVA revealed a significant Condition × Time interaction [*F*(4, 232) = 7.26, p < 0.001, $\eta_{p}^{\text{2}}$ = 0.088]. As shown in the inset of Fig 2, the value in the CD condition (0.095 ± 0.019 mM･mm) was statistically significantly higher than that in the PD condition (0.042 ± 0.016 mM･mm. *p* = 0.016) in the 9–10 min time-window block.

As shown in the inset, the average AoxyHb_F_ in the CD condition was significantly higher than that in the PD condition in the 9–10 min time-window block. *p < 0.05, Bonferroni-corrected pairwise comparison following the two-way repeated-measures ANOVA. Data are shown as the average ± SEM.

These results indicate that activity of MFPR during eating was changed depend on food, especially the late phase after the start of eating. The response lasted longer for the provided foods (CD) than for the meals selected by participants themselves (PD).

### The number of dislike foods associate with the activity of MFPR during “eating” the food

We performed PCA on the results of FFQg and questionnaire of food preference/food-intake behavior, for the purpose of dimensionality reduction in order to reduce Type I error. As shown in S2 Fig., S3 Fig., and S4 Fig., it was possible to classify the results of FFQg into seven new categories: Eat rice in the morning principal component score (PCS), Eat bread in the morning PCS (from cereal grains), Protein-balanced PCS, Meat PCS, Dairy products PCS (from protein-related foods), Vegetable-balanced PCS, and Not eat vegetables in the morning PCS (from vegetables). Also as shown in S5 Fig., it was possible to classify the results of questionnaire of food preference into three new categories: Meat prefer PCS, Fish/vegetables prefer PCS, and Sweet not prefer PCS. Moreover, as shown in S6 Fig., it was possible to classify the results of questionnaire of food-intake behavior into two new categories; Purchase and cooking PCS and Proactive on food PCS. Knowledge of seasonal foods and the number of the dislike foods were not included in the PCA.

Next, we performed multiple regression analysis to clarify the associations between AoxyHb_F_ during “just-looking”, “eating” and these factors. AoxyHb_F_ during “just-looking” was not statistically significantly associated with food-intake behavior (*R*^*2*^ = 0.272, *Adj. R*^*2*^ = 0.074, *F*(9,126) = 1.12, *p* = 0.36, n = 136). AoxyHb_F_ during “just-looking” was also not statistically significantly associated with food preference (*R*^*2*^ = 0.030, *Adj. R*^*2*^ = not estimated, *F*(6,123) = 0.64, *p* = 0.70, n = 130). Moreover, AoxyHb_F_ during “just-looking” was not statistically significantly associated with food-intake behavior/knowledge of seasonal foods (*R*^*2*^ = 0.013, *Adj. R*^*2*^ = not estimated, *F*(5,128) = 0.344, *p* = 0.89, n = 134).

AoxyHb_F_ during “eating” was not statistically significantly associated with food-intake behavior in all time-window block. (*p* > 0.22). In contrast, AoxyHb_F_ during “eating” was statistically significantly associated with food preference in the 3–4 min (*R*^*2*^ = 0.100, *Adj. R*^*2*^ = 0.056, *F*(6,123) = 2.27, *p* = 0.041, n = 130), and the 9–10 min time-window block (*R*^*2*^ = 0.115, *Adj. R*^*2*^ = 0.069, *F*(6,115) = 2.49, *p* = 0.027, n = 122). As shown in Table 1, the number of dislike foods was positively associated with increased AoxyHb_F_ during “eating” (*b* = 0.016, *SE* = 0.0052, *β* = 0.28, *t* = 3.11, *p* = 0.0023) in *t*he 3–4 min time-window *b*lock. The Fish/vegetables prefer PCS was also positively associated with increased AoxyHb_F_ during “eating” (*b* = 0.025, *SE* = 0.012, *β* = 0.18, *t* = 2.13, *p* = 0.035) in 3–4 min *t*ime-window *b*lock. Moreover, as shown in Table 2, the number of dislike foods was positively associated with increased AoxyHb_F_ during “eating” (*b* = 0.012, *SE* = 0.0051, *β* = 0.21, *t* = 2.33, *p* = 0.022) in *t*he 9–10 min time-window block. Gender was negatively associated with increased AoxyH*b*_F_ during “eating” (*b* = −0.066, *SE* = 0.028, *β* = −0.22, *t* = −2.35, *p* = 0.021) in *t*he 9–10 min time-window block.

**Table 1:** Multiple regression analysis of AoxyHb_F_ in the MFPR detected by fNIRS during the “eating” condition and preference of foods in the 3-4 min time-window block.

	*b*	*SE*	*β*	*t*-value	*p*-value
**PD dummy**	0.011	0.026	0.036	0.42	0.68
**Gender**	−0.011	0.028	−0.035	−0.38	0.71
**MP PCS**	0.0029	0.0099	0.026	0.29	0.77
**FVP PCS**	0.025	0.012	0.18	2.13	0.035*
**SNP PCS**	−0.0010	0.013	−0.0071	−0.082	0.93
**NDF**	0.016	0.0052	0.28	3.11	0.0023**

PD dummy (CD = 0, PD = 1); gender (male = 0, female = 1). PCS, principal component score; MP PCS, Meat prefer PCS; FVP PCS, Fish/vegetables prefer PCS, SNP PCS, Sweet not prefer PCS; NDF, Number of dislike foods. ** indicates *p* < 0.01, * indicates *p* < 0.05

**Table 2:** Multiple regression analysis of AoxyHb_F_ in the MFPR detected by fNIRS during the “eating” condition and preference of foods in the 9-10 min time-window block.

	*b*	*SE*	*β*	*t*-value	*p*-value
**PD dummy**	−0.051	0.026	−0.17	−1.96	0.052
**Gender**	−0.066	0.028	−0.22	−2.35	0.021*
**MP PCS**	−0.0031	0.0096	−0.022	−0.32	0.75
**FVP PCS**	0.016	0.012	0.12	1.37	0.17
**SNP PCS**	−0.0048	0.013	−0.034	−0.38	0.70
**NDF**	0.012	0.0051	0.21	2.33	0.022*

PD dummy (CD = 0, PD = 1); gender (male = 0, female = 1). PCS, principal component score; MP PCS, Meat prefer PCS; FVP PCS, Fish/vegetables prefer PCS; SNP PCS, Sweet not prefer PCS; NDF, Number of dislike foods. * indicates *p* < 0.05

Food-intake behavior/knowledge of seasonal foods was not significantly associated with AoxyHb_F_ during “eating” (p > 0.12) in the time-window blocks from 1–2 min to 7–8 min. In contrast, food-intake behavior/knowledge of seasonal foods was significantly associated with AoxyHb_F_ during “eating” in the 9–10 min time-window block (*R*^*2*^ = 0.10, *Adj. R*^*2*^ = 0.063, *F*(5,119) = 2.66, *p* = 0.026, n = 125). As shown in Table 3, however, gender (*b* = −0.055, *SE* = 0.027, *β* = −0.19, *t* = −2.03, *p* = 0.044) and *t*he PD dummy varia*b*le (*b* = −0.051, *SE* = 0.025, *β* = −0.17, *t* = −1.99, *p* = 0.049) were nega*t*ively associated with increased in AoxyH*b*_F_ during “eating” in the 9–10 min time-window *b*lock.

**Table 3:** Multiple regression analysis of AoxyHb_F_ in MFPR detected by fNIRS during the “eating” condition and food-intake behavior/knowledge of seasonal foods in the 9-10 min time-window block.

	*b*	*SE*	*β*	*t*-value	*p*-value
**PD dummy**	−0.051	0.024	−0.17	−1.99	0.049*
**Gender**	−0.055	0.027	−0.19	−2.03	0.044*
**PC PCS**	0.0083	0.010	0.081	0.83	0.41
**PF PCS**	0.0089	0.012	0.069	0.77	0.44
**KSF**	0.0038	0.0027	0.14	1.42	0.16

PC PCS, Purchase and cooking PCS; PF PCS, Proactive on food PCS; KSF, knowledge of seasonal foods. * indicates *p* < 0.05

These results indicated that activity of MFPR during “eating” was influenced by the number of dislike foods and gender. These differences were detected mainly in the later phase of eating.

### The knowledge of foods and gender were potentially associated with the number of dislike foods

Finally, we examined the relationship between the number of dislike foods and each factor in this study. For this purpose, participants were divided into those who had dislike foods (DF group, n = 46) and those who did not (NDF group, n = 22). As mentioned in Methods, four variables (gender, knowledge of seasonal foods, Purchase and cooking PCS, and Proactive on food PCS) exhibited potential associations in the univariable analysis (*p* < 0.1). However, because of the limited number of participants, we selected two variables (gender and knowledge of seasonal foods. *p* < 0.05) for the final multivariable binary logistic regression model. As shown in Table 4, the logistic regression model was statistically significant (Nagelkerke R^2^ = 0.445), explained 44.5% of the variance in the food preference, and correctly classified 87.0% of cases. After controlling for other variables, females were significantly more likely to have dislike foods than males [*B* = 2.23, *SE* = 0.75, *p* = 0.0029, *OR* (95% CI) = 9.26 (2.13-40.1)]. Knowledge of seasonal foods was also associated with a decreased likelihood of having dislike foods [*B* = -0.29, *SE* = 0.079, *p* < 0.001, *OR* (95% CI) = 0.75 (0.64-0.87)].

**Table 4:** Logistic regression analysis predicting the food preference.

Variables	*B*	*SE*	Wald	*p*-value	*OR* (95% CI)
**Gender** **(male = 0, female = 1)**	2.23	0.75	8.86	0.0029*	9.26 (2.13-40.1)
**Knowledge of seasonal foods**	−0.29	0.079	13.64	<0.001	0.75(0.64-0.87)

OR, odds ratio; CI, confidence interval. Model χ^2^ (2) = 26.05, p < 0.001; Nagelkerke R^2^ = 0.445

Taken together, knowledge of foods and gender were associated with preference of foods, and these factors may be associated with the activity of the MFPR during eating behavior. This study suggests that fNIRS measurement of brain function may provide preliminary information related to food preferences, and knowledge of foods.

## Discussion

In this study, we found that the activity of MFPR while “just-looking” or “eating” some dishes differed depending on the food preference and gender. In particular, food preference contributes to the activity of MFPR. MPFC is known to be involved in multiple functions, including value representation [25], reward processing [26], and self-referential processing [27]. In addition, appetite regulation is primarily mediated by subcortical regions such as the hypothalamus [28]. Therefore, appetite and related decision-making cannot be discussed solely on the basis of MFPR responses. Because short-separation channels were not used, superficial physiological signals and movement-related components may not have been fully removed in this study. However, although the number of experiments was limited and methodological improvements are needed, the present findings suggest that measuring MFPR responses during eating using fNIRS may be useful for estimating participants’ food preferences.

The activity of MPFC modulates behavior, including eating [9], and category-dependent preference signals have been found in the MPFC [29]. The activity of MPFC is also correlated with schematic prior knowledge and contributes to memory recall or understanding of future events [30,31]. This function may also be associated with eating behavior. Thus, knowledge of foods may be associated with the activity of MPFC during eating. As shown in Table 4, knowledge of seasonal foods was potentially associated with the preference of food. This factor may also indirectly modify the activity of MFPR during eating.

In this study, menstrual cycles were not recorded in order to reduce the psychological burden on female participants. However, menstrual cycles may have influenced the results of our study [32]. As reported previously, activity of MPFC induced by odor shows gender- and age- dependent differences [33]. Because we also found a gender-related difference in the activity of MFPR during eating, we cannot exclude the possibility that menstrual cycle-related changes influenced food preference or fNIRS responses. Further studies will be necessary in the future.

In this study, the CD was prepared by the researchers, whereas the PD was freely selected and purchased by each participant. As a result, the type of food, nutritional composition, taste, appearance, and temperature were not standardized across conditions. The amount of the CD was selected for feasibility in future studies of older adults, but it may have been insufficient for some young male participants. Changes in the concentration of blood sugar may have been associated with the MFPR activity in this study. This is another limitation of this research, and we plan to address it by improving the methodology in the future.

The next step of this study will be performed on elderly participants. However, there are still several concerns. Olfactory, taste, and visual functions decrease with aging [34–36]. For example, the activity of MPFC during an olfactory task was found to decrease with aging [33]. Thus, we should expand the age range of participants such as middle age (around 40 years old) and early elderly age (60 years old without appetite loss) before observing the main target age group (over 80 years old with appetite loss). Moreover, in this study, the participants ate whole dishes, but which are sometimes difficult for elderly people to eat reasons such as the amount of foods is large, swallowing difficulty, and the time course of testing. This study found that the MFPR responses induced by CD and PD differed from 9 min after the start of eating. This result is thought to reflect the difficulty of measuring brain function while participants consume multiple foods, because long-duration recording is required. On the other hand, the main significant result (the association between the number of dislike foods and the activity of MFPR during eating) was also detected from 3–4 min after the start of eating (although CD and PD did not differ significantly in this time-window block). Future studies could use a limited set of standardized test foods, such as small rice balls or pureed foods.

## Conclusion

Although fNIRS has limited spatial resolution and its signals reflecrt a mixture of multiple brain functions, observing MFPR activity during eating using fNIRS may eventually help estimate food preferences associated with participants’ knowledge of foods and gender, potentially including among elderly. Using fNIRS in combination with food intake tests may contribute to the treatment of food-related disorders (such as poor appetite in older adults) across many age groups, including elderly. However, further technological improvements will be needed to determine whether this approach has definitive clinical significance.

## Supporting information

S1 Table Food preference questionnaire. All written in Japanese. Note: ④ Japanese jelly-like food made from starch. ⑨ Japanese food made from tofu. ㉑ Japanese mustard spinach. ㊱Japanese mushroom. ㊲Japanese mushroom. ㊳Japanese mushrooms and also used in Chinese dishes. ㊹ Japanese stick-like baked or steamed fishcake. The “hot” taste refers to the “like taste of chili pepper” in this study, and we did not use the term “spicy”. Japanese usually associate “spicy” with the taste of other spices. Thus, if participants answered that they like the hot taste, they meant that they like the chili pepper taste. The numbers in parentheses are the numbers replaced during PCA and are not shown to the subjects.
(DOCX)

S2 Table Questionnaire on food-intake behavior/knowledge of food. All written in Japanese. The numbers in parentheses are the numbers replaced during PCA and are not shown to the subjects.
(DOCX)

S1 Text Appendix of control dish (CD; typical Japanese home-cooked meal). (DOCX)

S1 Fig Experimental design of food intake test and NIRS recording. (A) Schematic of probes positions. We used signals from cHs 7–10 as activity in the medial/frontopolar prefrontal regions (MFPR). (B) Control dish (CD; typical Japanese home-cooked meal) in this study. (C) Experimental flow. After attachment of the fNIRS probes, 20 s was allocated for baseline recording (participants were instructed to relax and wait), 20 s for “just looking”, and sufficient time for “eating” (the first 10 min was used for analysis).
(TIF)

S2 Fig Principal component analysis (PCA) of questionnaire (cereal grains). Results of PCA of cereal grains from the FFQg (eating rice, bread, and, noodles in the morning, noon, and, evening). PC1(50.2%) and PC2(26.3%) together explained 76.5% of the total variance. The Kaiser-Meyer-Olkin (KMO) measure of sampling adequacy was 0.605, and Barlett’s test yielded an approximate χ^2^ = 11.53, *p* = 0.009.
(TIF)

S3 Fig PCA of questionnaire (protein-related foods). Results of PCA of protein-related foods from the FFQg (eating meat, fish, and, soybeans in the morning, noon, and, evening; eating dairy products and eggs). PC1 (33.4%), PC2 (17.4%), and PC3 (13.2%) together explained 64.0% of the total variance. The KMO measure of sampling adequacy was 0.691, and Barlett’s test yielded an approximated χ^2^ = 149.48, *p* < 0.001.
(TIF)

S4 Fig PCA of questionnaire (vegetable). Results of PCA of vegetables from the FFQg (eating green and yellow vegetables and light-colored vegetables in the morning, noon, and, evening; eating fruits). PC1 (58.4%) and PC2 (16.2%) together explained 74.6% of the total variance. The KMO measure of sampling adequacy was 0.581, and Bartlett’s test yielded an approximated χ^2^ = 196.43, *p* < 0.001.
(TIF)

S5 Fig PCA of questionnaire (food preference). Results of PCA of the food preference questionnaire (eleven factors from Supplemental Table 1, item 1–3). PC1 (27.8%), PC2 (18.7%), and PC3 (15.9%) together explained 62.5% of the total variance. The KMO measure of sampling adequacy was 0.619, and Bartlett’s test yielded an approximated χ^2^ = 42.12, *p* = 0.004.
(TIF)

S6 Fig PCA of questionnaire (food-intake behavior/knowledge of food). Results of PCA of the questionnaire on food-intake behavior/knowledge of food (from Supplemental Table 2, items 2–1, 2–2, 2–4, 2–5, 2–6, and 2–7). PC1 (41.5%) and PC2 (24.9%) together explained 66.4% of the total variance. The KMO measure of sampling adequacy was 0.618, and Bartlett’s test yielded an approximated χ^2^ = 60.93, *p* < 0.001.
(TIF)
