# Supplementary material for: Food preference and gender are associated with medial/frontopolar prefrontal regions functional near-infrared spectroscopy responses during eating: An exploratory study in young adults
Source: PLoS One. 2026 Aug 3;21(8):e0343481. doi: 10.1371/journal.pone.0343481 (PMC13432127; doi:10.1371/journal.pone.0343481)
Supplement: S1 Table — All written in Japanese. Note: ④ Japanese jelly-like food made from starch. ⑨ Japanese food made from tofu. ㉑ Japanese mustard spinach. ㊱Japanese mushroom. ㊲Japanese mushroom. ㊳Japanese mushrooms and also used in Chinese dishes. ㊹ Japanese stick-like baked or steamed fishcake. The “hot” taste refers to the “like taste of chili pepper” in this study, and we did not use the term “spicy”. Japanese usually associate “spicy” with the taste of other spices. Thus, if participants answered that they like the hot taste, they meant that they like the chili pepper taste. The numbers in parentheses are the numbers replaced during PCA and are not shown to the subjects. (DOCX) [file pone.0343481.s001.docx]

**Supplemental Table 1 Food preference questionnaire**

| **Questionnaire** | **Answers** |
| --- | --- |
| 1-1. Are there any foods you currently disliked or don't want to eat, for reasons other than allergies? | A) Yes　B) No　C) I don’t know |
| 1-2. Mark all the foods you dislike and don't want to eat. | ①Udon ②Cooked rice ③Bread ④Konnyaku ⑤Sweet potato ⑥potato ⑦Red beans ⑧Soy ⑨Koya-tofu ⑩Tofu ⑪Fried tofu ⑫Sesame ⑬Pumpkin ⑭Green peas ⑮Green beans ⑯Carrot ⑰Green onion ⑱Green pepper ⑲Broccoli ⑳Spinach ㉑Komatsuna ㉒Cabbage ㉓Cucumber ㉔Burdock ㉕Japanese white radish ㉖Onion ㉗Corn ㉘Eggplant ㉙Chinese cabbage ㉚Tomato ㉛Mini tomato ㉜Banana ㉝Tangerine ㉞Apple ㉟Pineapple ㊱Enoki mushroom㊲Shimeji mushroom㊳Dried shiitake mushroom㊴Dried seaweed ㊵Hijiki seaweed ㊶Wakame seaweed ㊷Squid ㊸Shrimp ㊹Chikuwa ㊺Spanish mackerel ㊻Salmon ㊼Liver ㊽Beef ㊾Chicken ㊿Pork Cheese Yogurt Milk Egg Quail eggs Others (write as much as you like) |
| 1-3. Tastes in the seasonings in foods you usually eat and what you are careful about. | A) High (=3) B) moderately (=2) C）low (=1)  （salty, sweet, sour, bitter, hot）  A) like(=3) B) neutral (=2) C）dislike (=1)  （carbohydrates, oil, protein (meat), protein (fish), vegetables, alcohol） |
| Frequency | a）Don’t care b）Try to refrain from eating c）Concerned but refrain from eating |
| （Alcoholic drink） | A) Every day B) 3–5 times/week　C）1–2 times/week D) less than once/month (occasional drinking）  ・How many glasses/occasion ?(free writing) |
| 1-4. Cigarettes | A) Smoke B) Not Smoke　C）Previously smoked but stopped  ・How many cigarettes per day? (free writing) |
| Frequency | A）Don’t care B）Try to refrain from smoking C）Concerned but refrain from smoking |

All written in Japanese.

Note: ④ Japanese jelly-like food made from starch. ⑨ Japanese food made from tofu. ㉑ Japanese mustard spinach. ㊱Japanese mushroom. ㊲Japanese mushroom. ㊳Japanese mushrooms and also used in Chinese dishes. ㊹ Japanese stick-like baked or steamed fishcake.

The “hot” taste refers to the “like taste of chili pepper” in this study, and we did not use the term “spicy”. Japanese usually associate “spicy” with the taste of other spices. Thus, if participants answered that they like the hot taste, they meant that they like the chili pepper taste.

The numbers in parentheses are the numbers replaced during PCA and are not shown to the subjects.

**Supplemental Table 2** **Questionnaire on food-intake behavior/knowledge of food**

| **Questionnaire** | **Answers** |
| --- | --- |
| 2-1. Do you eat three meals a day? | A）Eat three meals every day (=3)　B) Eat three meals half of the week (=2) C) Mainly do not eat three meals (=1) |
| When do you most often skip meals?  (→omitted at PCA) | A) Never skip　B) Breakfast　C) 　Lunch　D) Dinner |
| 2-2. Do you eat a late-night snack (within 2 hours before going to bed)? | A) Never eat (=1)　B) Once per week (=2) C) More than three times per week (=3) |
| 2-3. Have you ever gone on a diet that restricted certain foods, such as eliminating carbohydrates? (→omitted at PCA) | 1. Ongoing　B) Previously　C) Never |
| 2-4. Do you ever cook your own food? | A) More than three days per week (=4)　B) Once per week (=3)　C) Hardly ever cooked (=2) D) Don't know how to cook(=1) |
| 2-5. Do you ever go to a new and popular restaurants? | A) Often visit (=3)　B) Sometimes visit (=2)　C) Not visit/interested (=1) |
| 2-6. Do you watch/listen to cooking-related programs/content (regardless of the media)? | A) Watch/listen five or more times per week (=3)　B) Watch/listen once or twice per week (=2)　C) Not watch/listen/interested (=1) |
| Do you test the information obtained from those programs and contents? | A) Try often (try making/tries to eat)(=3) B) Sometimes try (=2)　C) Just watch/listen (=1) |
| 2-7. Do you ever buy ingredients (food that cannot be eaten without cooking) on ​​your own at a supermarket? | A) Often (=4)　B) Sometimes (=3)　C) Buy if asked (=2)　D) I've never bought one/I don't know how to choose one (=1) |
| 2-8. Please list as many of the following ingredients as you can think of (up to five) |  |
| Ingredients that are in season in spring | free writing |
| Ingredients that are in season in summer | free writing |
| Ingredients that are in season in autumn | free writing |
| Ingredients that are in season in winter | free writing |

All written in Japanese.

The numbers in parentheses are the numbers replaced during PCA and are not shown to the subjects.
