# Supplementary material for: Food preference and gender are associated with medial/frontopolar prefrontal regions functional near-infrared spectroscopy responses during eating: An exploratory study in young adults
Source: PLoS One. 2026 Aug 3;21(8):e0343481. doi: 10.1371/journal.pone.0343481 (PMC13432127; doi:10.1371/journal.pone.0343481)
Supplement: S2 Table — All written in Japanese. The numbers in parentheses are the numbers replaced during PCA and are not shown to the subjects. (DOCX) [file pone.0343481.s002.docx]

**Supplemental Table 2** **Questionnaire on food-intake behavior/knowledge of food**

| **Questionnaire** | **Answers** |
| --- | --- |
| 2-1. Do you eat three meals a day? | A）Eat three meals every day (=3)　B) Eat three meals half of the week (=2) C) Mainly do not eat three meals (=1) |
| When do you most often skip meals?  (→omitted at PCA) | A) Never skip　B) Breakfast　C) 　Lunch　D) Dinner |
| 2-2. Do you eat a late-night snack (within 2 hours before going to bed)? | A) Never eat (=1)　B) Once per week (=2) C) More than three times per week (=3) |
| 2-3. Have you ever gone on a diet that restricted certain foods, such as eliminating carbohydrates? (→omitted at PCA) | 1. Ongoing　B) Previously　C) Never |
| 2-4. Do you ever cook your own food? | A) More than three days per week (=4)　B) Once per week (=3)　C) Hardly ever cooked (=2) D) Don't know how to cook(=1) |
| 2-5. Do you ever go to a new and popular restaurants? | A) Often visit (=3)　B) Sometimes visit (=2)　C) Not visit/interested (=1) |
| 2-6. Do you watch/listen to cooking-related programs/content (regardless of the media)? | A) Watch/listen five or more times per week (=3)　B) Watch/listen once or twice per week (=2)　C) Not watch/listen/interested (=1) |
| Do you test the information obtained from those programs and contents? | A) Try often (try making/tries to eat)(=3) B) Sometimes try (=2)　C) Just watch/listen (=1) |
| 2-7. Do you ever buy ingredients (food that cannot be eaten without cooking) on ​​your own at a supermarket? | A) Often (=4)　B) Sometimes (=3)　C) Buy if asked (=2)　D) I've never bought one/I don't know how to choose one (=1) |
| 2-8. Please list as many of the following ingredients as you can think of (up to five) |  |
| Ingredients that are in season in spring | free writing |
| Ingredients that are in season in summer | free writing |
| Ingredients that are in season in autumn | free writing |
| Ingredients that are in season in winter | free writing |

All written in Japanese.

The numbers in parentheses are the numbers replaced during PCA and are not shown to the subjects.
