# Supplementary material for: Food preference and gender are associated with medial/frontopolar prefrontal regions functional near-infrared spectroscopy responses during eating: An exploratory study in young adults
Source: PLoS One. 2026 Aug 3;21(8):e0343481. doi: 10.1371/journal.pone.0343481 (PMC13432127; doi:10.1371/journal.pone.0343481)
Supplement: S1 Text — (DOCX) [file pone.0343481.s003.docx]

**Appendix**

**Breakfast (only estimated. Participants did not eat this)**

Pizza toast, salad, banana yogurt

**Lunch (eaten as CD)**

Rice, grilled pork with ginger, cabbage, seaweed and tuna salad, tofu miso soup, Japanese pear (or Apple)

**Dinner (only estimated. Participants did not eat this)**

Rice, Chinese style of eight kinds of vegetables, Chinese stir-fried spinach, Chinese style corn soup, sweet potato and apple honey butter

We used only the lunch menu for the experiment

**Details of the lunch.**

| Ingredients |  | Quantity |
| --- | --- | --- |
| Rice |  | 150 g |
| Grilled pork with ginger | Pork | 70 g |
|  | Sugar | 2 g |
|  | Soy sauce | 3 g |
|  | Cabbage | 30 g |
| Seaweed and tuna salad | Broccoli | 20 g |
|  | Dried sea weed | 0.5 g |
|  | Tuna | 10 g |
|  | Tomato | 20 g |
|  | Dressing | 10 g |
| Tofu miso soup | Tofu | 20 g |
|  | Japanese radish | 20 g |
|  | Onion | 5 g |
|  | Miso | 6 g |
|  | Dashi | 150 g |
| Japanese Pear (or Apple) |  | 30 g |

PD depended on participants and thus, we did not control the quantity of each food. Most of all participants purchased variety of lunch box, rice balls, and sandwiches.
