# Supplementary figures and images for: Food preference and gender are associated with medial/frontopolar prefrontal regions functional near-infrared spectroscopy responses during eating: An exploratory study in young adults
Source: PLoS One. 2026 Aug 3;21(8):e0343481. doi: 10.1371/journal.pone.0343481 (PMC13432127; doi:10.1371/journal.pone.0343481)

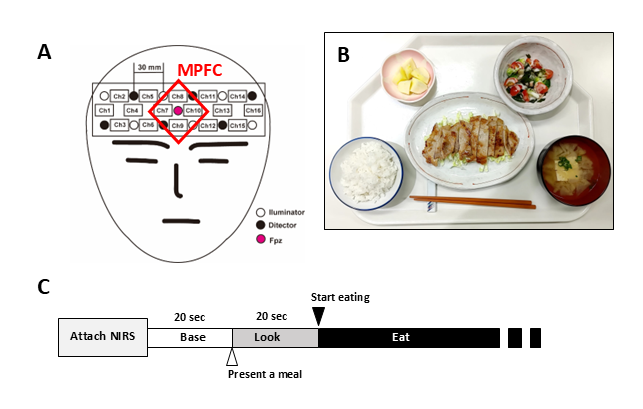

Supplement: S1 Fig — (A) Schematic of probes positions. We used signals from cHs 7–10 as activity in the medial/frontopolar prefrontal regions (MFPR). (B) Control dish (CD; typical Japanese home-cooked meal) in this study. (C) Experimental flow. After attachment of the fNIRS probes, 20 s was allocated for baseline recording (participants were instructed to relax and wait), 20 s for “just looking”, and sufficient time for “eating” (the first 10 min was used for analysis). (TIF) [file pone.0343481.s004.TIF]

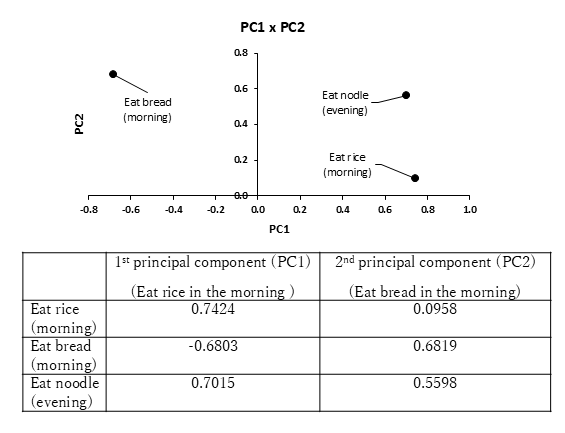

Supplement: S2 Fig — Results of PCA of cereal grains from the FFQg (eating rice, bread, and, noodles in the morning, noon, and, evening). PC1(50.2%) and PC2(26.3%) together explained 76.5% of the total variance. The Kaiser-Meyer-Olkin (KMO) measure of sampling adequacy was 0.605, and Barlett’s test yielded an approximate χ2 = 11.53, p = 0.009. (TIF) [file pone.0343481.s005.TIF]

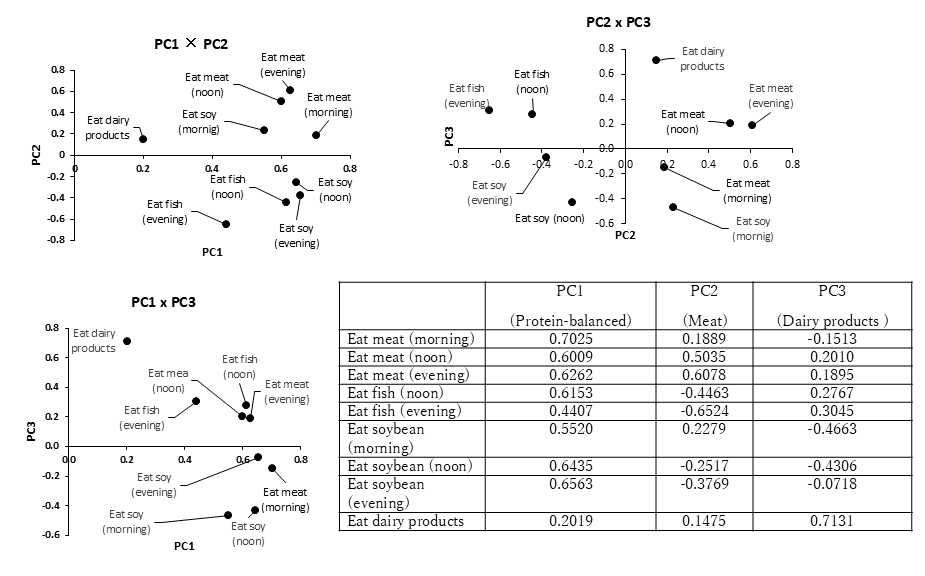

Supplement: S3 Fig — Results of PCA of protein-related foods from the FFQg (eating meat, fish, and, soybeans in the morning, noon, and, evening; eating dairy products and eggs). PC1 (33.4%), PC2 (17.4%), and PC3 (13.2%) together explained 64.0% of the total variance. The KMO measure of sampling adequacy was 0.691, and Barlett’s test yielded an approximated χ2 = 149.48, p < 0.001. (TIF) [file pone.0343481.s006.TIF]

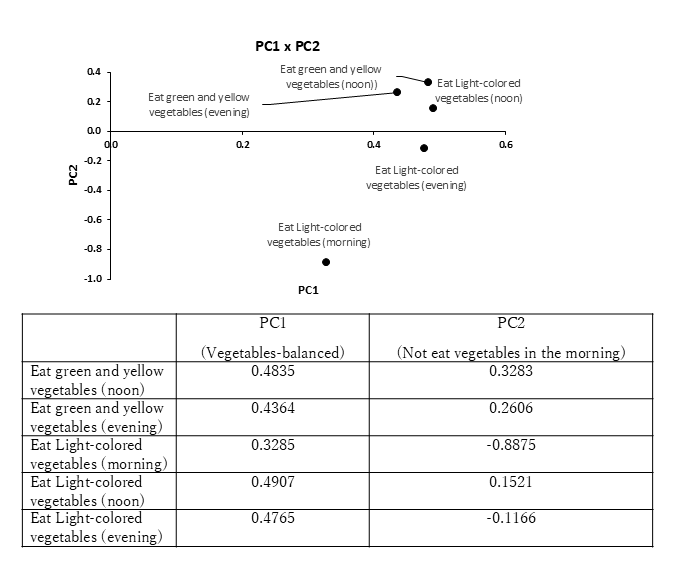

Supplement: S4 Fig — Results of PCA of vegetables from the FFQg (eating green and yellow vegetables and light-colored vegetables in the morning, noon, and, evening; eating fruits). PC1 (58.4%) and PC2 (16.2%) together explained 74.6% of the total variance. The KMO measure of sampling adequacy was 0.581, and Bartlett’s test yielded an approximated χ2 = 196.43, p < 0.001. (TIF) [file pone.0343481.s007.tif]

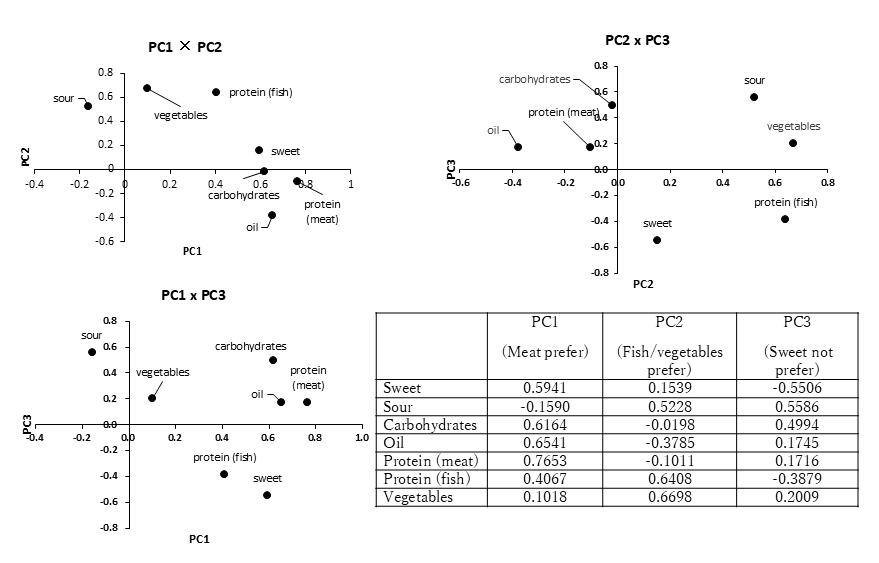

Supplement: S5 Fig — Results of PCA of the food preference questionnaire (eleven factors from Supplemental Table 1, item 1–3). PC1 (27.8%), PC2 (18.7%), and PC3 (15.9%) together explained 62.5% of the total variance. The KMO measure of sampling adequacy was 0.619, and Bartlett’s test yielded an approximated χ2 = 42.12, p = 0.004. (TIF) [file pone.0343481.s008.TIF]

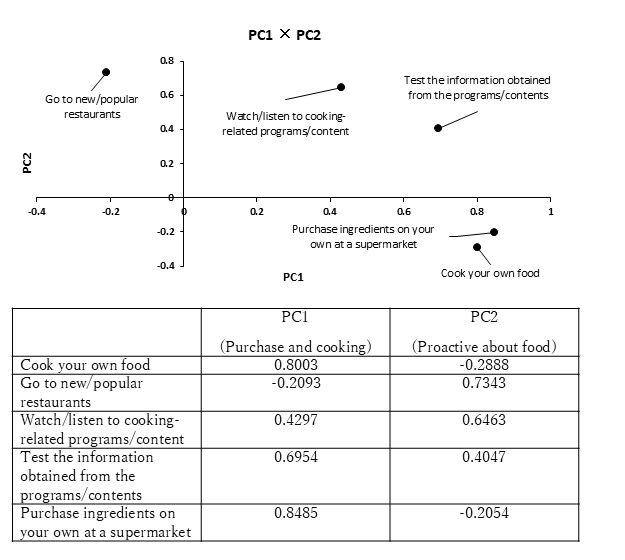

Supplement: S6 Fig — Results of PCA of the questionnaire on food-intake behavior/knowledge of food (from Supplemental Table 2, items 2–1, 2–2, 2–4, 2–5, 2–6, and 2–7). PC1 (41.5%) and PC2 (24.9%) together explained 66.4% of the total variance. The KMO measure of sampling adequacy was 0.618, and Bartlett’s test yielded an approximated χ2 = 60.93, p < 0.001. (TIF) [file pone.0343481.s009.TIF]
