# Supplementary material for: Food preference and gender are associated with medial/frontopolar prefrontal regions functional near-infrared spectroscopy responses during eating: An exploratory study in young adults
Source: PLoS One. 2026 Aug 3;21(8):e0343481. doi: 10.1371/journal.pone.0343481 (PMC13432127; doi:10.1371/journal.pone.0343481)
Supplement: Attachment — Submitted filename: Reviewer.docx [file pone.0343481.s011.docx]

*Reviewer #1: This manuscript addresses an interesting topic and has potential clinical implications. However, several methodological and interpretational issues should be addressed before publication.*

*① The stated aim of this study is to understand the mechanism of food-related decision-making in the elderly. However, the experimental task only required participants to “look at” or “eat” food, and did not include any actual decision-making process such as choosing between alternative foods. Therefore, interpreting the observed MPFC activity as reflecting food-related decision-making is not fully supported by the experimental design.*

As reviewers 1 and 3 pointed out, results of this study not enough to elucidate the decision-making mechanisms involved in looking/eating foods. This is because the decision-making mechanisms are complex, and the spatial resolution of NIRS is not sufficient. As mentioned in below, one of our aims of this study is to clarify the relationship between activity in the food-dependent MPFC and food preferences and consumption frequency in young healthy adults, with the aim of establishing methods for assessing food preferences and appetite in elderly individuals with poor appetite. Thus, we revised the manuscript for focus on this point (Abstract, P3L24-28, Introduction, P7L79-82, L83-87).

*② In this study, the control dish (CD) was prepared by the researchers, whereas the preferred dish (PD) was freely selected and purchased by each participant. As a result, the type of food, nutritional composition, taste, appearance, and temperature were not standardized across conditions. Consequently, it is difficult to determine whether the observed differences in MPFC activity reflect food preference or simply differences in the physical and sensory properties of the foods.*

The CD amount was selected for feasibility in future studies of older adults, but it may have been insufficient for some young male participants. Changing the concentration of blood sugar may potentially associate with the results of the MFPR activity in this study. This is also one of the limitations of this research, and we plan to address it by improving the methodology in the future. We mentioned it in Discussion (P29L387-L394 and P30L403-410).

*③ While the amount of the control dish (CD) was standardized based on caloric intake, the preferred dish (PD) was only required to be “an amount that can be completely consumed,” without controlling for caloric content or portion size. Since satiety and metabolic states such as blood glucose levels can influence brain activity, this lack of control may represent an important confounding factor.*

This is also the important point to concern. Blood glucose level can influence the brain activity. To clarified the point, for example, study should be conducted combining continuous blood glucose measurement methods using such as Freestyle Libre (Abbott). To avoid the effect of fullness, we analyzed the data of eating for first ten min in this study. However, this time setting also not established. We plan to modified the methods in future study. We mentioned it in the Discussion (P29L387-L394 and P30L403-410).

*④ Participants were classified as responders when the average z-score exceeded 2. However, the rationale for this threshold is not clearly justified. Given that fNIRS signals are subject to substantial inter-individual variability and noise, the use of a single arbitrary threshold for classification may influence the interpretation of the results.*

We removed the responder/non-responder classification and analyzed continuous baseline-corrected oxyHb values. We also not made threshold of activity to reduce the Type I error. We mentioned it in Methods (P16L219-P17L230).

*⑤ Although the total sample size was 68 participants, dividing them into four groups resulted in relatively small sample sizes for each group. Moreover, multiple statistical tests were conducted across numerous questionnaire variables. This raises concerns about the inflation of Type I error due to multiple comparisons.*

Thank you very much for your kind suggestion. We used PCA to reduce dimensionality and report the analyses as exploratory. We hope this modification reduce the concern of reviewer 1.

As shown in revised manuscript, preference of food and gender significantly associated with the amplitude of oxyHb induced by food intake. The knowledge of seasonal foods potentially associated with the preference of foods and thus, we may observe these factors by using fNIRS. Although the methodology need establishing (e.g., what foods appropriate to use as a test food, detecting other factors such as eye movement), we think that fNIRS is useful for detecting some food choice rerating behavior of human (Results of revised manuscript. P18L241-P26L357, Tables 1 to 4).

*⑥ The ultimate goal of the study is to understand appetite loss in the elderly; however, the experiments were conducted only in young participants. Since aging is associated with changes in taste, olfaction, cognitive function, and neural activity patterns, caution is required when generalizing the findings from young adults to elderly populations.*

As mentioned in discussion, results of this study could not generalize to elderly people because of the change in taste, olfaction, cognitive function and neural activity patterns. However, at least we found the recording of fNIRS during eating food can estimate some food dependent habit and behavior, and thus, modifying the method and observing the response from medial/frontopolar prefrontal region in elderly could be make sense. It is future study. We mentioned it in Discussion (P30L395-P31L410).

*⑦ Continuous fNIRS signals were dichotomized into responder and non-responder groups. Such dichotomization may lead to loss of information and reduced statistical power.*

As mentioned above, we performed multiple regression analysis directly on response of fNIRS without grouping (Results of revised manuscript. P18L241-P26L357, Tables 1 to 4).

*⑧ In this study, signals recorded around the Fpz region (cH7–10) were interpreted as MPFC activity. However, due to the relatively low spatial resolution of fNIRS and probe placement, the measured signals may reflect broader prefrontal activity, including the frontopolar cortex (BA10), rather than specific MPFC subregions. In addition, fNIRS cannot adequately measure deeper cortical regions such as the ventromedial prefrontal cortex. Furthermore, eating tasks involve chewing, facial muscle activity, and superficial blood flow changes, which may contaminate the signals. Although motion-related components were removed using a mathematical approach, short-separation channels were not used to remove superficial signals. Therefore, caution is required when interpreting these signals as direct indicators of MPFC activity.*

Thank you very much for your helpful suggestion. We now refer to these as fNIRS signals recorded over the medial/frontopolar prefrontal region. We mentioned it in Methods (P14L194-P15L200) and Discussion (P28L362-372 and P31L413-416).

*⑨ The study interprets MPFC activity as reflecting food-related decision-making and appetite-related processes. However, the MPFC is known to be involved in multiple functions, including value representation, reward processing, and self-referential processing. In addition, appetite regulation is primarily mediated by subcortical regions such as the hypothalamus. Therefore, attributing the observed MPFC activity specifically to decision-making or appetite regulation may be an overinterpretation, and these findings should be interpreted with caution.*

As mentioned by reviewer 1, we revised the manuscript to avoid interpreting the signals as direct indicators of decision-making or appetite regulation. We hope this revise reduced the concern of reviewer 1. We mentioned it in Discussion (P28L362-372)

*Reviewer #2: Congratulations , I enjoy reading your interested paper.*

*As you recommended in your paper, I agree with further technical improvements culd be required in order to determine whether the food intake test should be combined with fNIRS to contribute to the treatment of disorders in the elderly with TCA.*

*Also I completely agree to study younger people around 40 years old.*

Thank you for your comments. We plan to refine the method and then test it in middle-aged and older adults in future studies.

*Reviewer #3: This study used functional near-infrared spectroscopy to investigate the relationship between activity in the food-dependent medial prefrontal cortex (MPFC) and food preferences and consumption frequency; its purpose and methods are quite interesting. However, the explanation of the objectives and methods used to test the research hypothesis appears to be unclear. To improve the quality of the manuscript, I have pointed out the following major and minor issues:*

*Major　comments*

*Abstract and Introduction*

*I would appreciate it if you could clarify the true purpose of this study. The abstract states that　“to clarify the relationship of food dependent medial prefrontal cortex (MPFC) activity with food preference and intake frequency by functional near-infrared spectroscopy (fNIRS) to determine ways to treat the appetite loss and have difficulty in explain what they want to eat.” On the other hand, introduction states that “to clarify the relationship of the activity of MPFC with food intake by fNIRS to find satisfactory methods of mechanism of food eating decision-making in the elderly who have lost their appetite.”*

So sorry about our unestablished writing. We revised the Abstract and Introduction to state that this study examined young healthy adults as a methodological and exploratory step toward future studies in older adults. (Abstract, P3L24-28, Introduction, P7L79-82, L83-87).

*P7L82 The statement mentions “to identify satisfactory methods for understanding the mechanisms underlying food-related decision-making.” Does the research design used in this study—which employs regression analysis to identify food preferences and eating habits that are more likely to vary between responders and non-responders—align with the objective of elucidating decision-making mechanisms? Please confirm whether the research question and the design are consistent.*

What we wanted to do in this study was to examine whether fNIRS could be used as a biosensor for appetite, usual dietary habits, and preferences. For this purpose, initially, we planned to discriminate the responses of fNIRS using a pattern of responder/nonresponder based on z-scores. However, as pointed out by reviewer 1 and you, there was a risk that the selection of the z-score threshold would be unclear. Therefore, in the revised manuscript, we changed the method to look at the regression between the actual oxyHb values and the explanatory variables.

As shown in revised manuscript, preference of food and gender significantly associated with the amplitude of oxyHb induced by food intake. The knowledge of seasonal foods potentially associated with the preference of foods and thus, we may observe these factors by using fNIRS. Although the methodology need establishing (e.g., what foods appropriate to use as a test food, detecting other factors such as eye movement), we think that fNIRS is useful for detecting some food choice rerating behavior of human (Results of revised manuscript. P18L241-P26L357, Tables 1 to 4).

*P7L81 Since the participants in this study were not elderly individuals—but rather young healthy adults selected to establish the methodology—wouldn’t the purpose be “to clarify the relationship between activity in the food-dependent medial prefrontal cortex (MPFC) and food preferences and consumption frequency in young healthy adults, with the aim of establishing methods for assessing food preferences and appetite in elderly individuals with poor appetite”?*

Thank you very much for your kind suggestion. We mentioned as above, we revised the aim of this study “to clarify the relationship between activity in the food-dependent medial/frontopolar prefrontal regions (MFPR) and food preferences and consumption frequency in young healthy adults, with the aim of establishing methods for assessing food preferences and appetite in elderly individuals with poor appetite using functional near-infrared spectroscopy (fNIRS)” through over (Abstract, P3L24-28, Introduction, P7L79-82, L83-87).

*P10L132 Although this study aims to establish a methodology using young male and female participants, please explain why the energy intake was set at 34% of the daily energy intake of 1,600 kcal for elderly women. For example, a 544-kcal meal might be too small for young men, potentially leading to an underestimation of their responses.*

In this study, as in the conditions of PD, the aim is to conduct the experiment with an amount that can be fully consumed. To make it applicable to the elderly in the future, we set the amount of CD even older women can finish eating. As you pointed out, for example, young men may have reactions due to it being insufficient. For that reason, we analyzed it at 10 minutes after the start of eating to exclude response changes due to fullness. Issues regarding the amount of CD are planned to be addressed as improvements in methodology in the future. We mentioned it in Discussion (P29L387-P30L394)

*P9L109 While some studies suggest that dietary preferences and eating behaviors in females fluctuate with their menstrual cycle (ex. PMID: 38342313), were the menstrual cycles of the female participants standardized during the measurement period of this study? If there is no consistency, is it possible to exclude the possibility that periodically fluctuating food preferences influence activity in the medial prefrontal cortex?*

As mentioned by reviewer 3, menstrual cycles certainly affect the results of our study. As reported previously, activity of MPFC induced by odor shows gender and age dependency (Takatsuru et al., 2022). Because menstrual cycle phase was not recorded, we cannot exclude its possible influence and have added this as a limitation. We have added this as a limitation in Discussion (P29L380-386).

*Throughout this discussion*

*This study revealed that MPFC activity when just looking at specific foods or when eating them varies depending on food preferences, meal frequency, and knowledge about food. Please explain the academic or clinical significance of these results in terms of establishing the methods for assessing food preferences and appetite that this study aims to develop.*

Although NIRS has insufficient spatial resolution and its signals are a mixture of multiple brain functions, observing MPFC activity during eating using fNIRS may allow for estimation of food preferences and intake frequency in participants (may including the elderly). Using fNIRS as a biosensor in combination with food intake tests may contribute to the treatment of food-related disorders (such as anorexia nervosa) in many age groups, including the elderly. However, further technological improvements will be needed to determine whether it has definitive clinical significance. We mentioned it in Conclusion of Discussion (P31L413-419)

*Minor comments*

*P15L207 Since ANOVA is an abbreviation, please include (ANOVA) after the full name.*

Thank you for your suggestion. We revised the part (P15L212-214).

*P15L199 When distinguishing between responders and non-responders, a Z-score of more than 2 is often used; however, please clearly state the rationale for this cutoff to ensure readers can easily understand it.*

As mentioned above, threshold of z-score was depended on the change in z-score during baseline recording. Almost all z-score during baseline was ±2. It is well known that fNIRS responses contain a lot of individual differences and noise, and based on the purpose of this study (to clarify whether fNIRS can be used as a biosensor to estimate subjects' food preferences and eating behavior), we deliberately analyzed the measurements oxyHb after simple baseline correction, with the intention of providing a foundation for future research (Methods of revised manuscript, P14L191-194).

*P35 In the CD-responders post titled “Eat bread at breakfast,” there is an extra parenthesis in the SE section, so please correct it.*

Thank you for your suggestion. However, since the Results part totally revised, the part was disappeared in revised manuscripts.

*P39-42 Please check Table 4, as it may be duplicated.*

Thank you for your suggestion. However, since also the Tables part totally revised, the part was disappeared in revised manuscripts. We carefully checked to avoid duplication.
