# Supplementary material for: Food preference and gender are associated with medial/frontopolar prefrontal regions functional near-infrared spectroscopy responses during eating: An exploratory study in young adults
Source: PLoS One. 2026 Aug 3;21(8):e0343481. doi: 10.1371/journal.pone.0343481 (PMC13432127; doi:10.1371/journal.pone.0343481)
Supplement: Peer review history

# Peer review: Decision Letter 0

**DOI:** 10.1371/journal.pone.0343481.r001

**Authors:** Yoko Hasegawa

6 Apr 2026

PONE-D-26-06160

Food intake habits and preference affects the activity of medial frontal cortex during eating; functional near-infrared spectroscopy is potential biosensor for appetite study.

PLOS One

Dear Dr. Takatsuru,

Thank you for submitting your manuscript to PLOS ONE. After careful consideration, we feel that it has merit but does not fully meet PLOS ONE’s publication criteria as it currently stands. Therefore, we invite you to submit a revised version of the manuscript that addresses the points raised during the review process.

Please submit your revised manuscript by May 21 2026 11:59PM. If you will need more time than this to complete your revisions, please reply to this message or contact the journal office at plosone@plos.org. When you're ready to submit your revision, log on to https://www.editorialmanager.com/pone/ and select the 'Submissions Needing Revision' folder to locate your manuscript file.

Please include the following items when submitting your revised manuscript:

- A letter that responds to each point raised by the academic editor and reviewer(s). You should upload this letter as a separate file labeled 'Response to Reviewers'.
- A marked-up copy of your manuscript that highlights changes made to the original version. You should upload this as a separate file labeled 'Revised Manuscript with Track Changes'.
- An unmarked version of your revised paper without tracked changes. You should upload this as a separate file labeled 'Manuscript'.

If you would like to make changes to your financial disclosure, please include your updated statement in your cover letter. Guidelines for resubmitting your figure files are available below the reviewer comments at the end of this letter.

If applicable, we recommend that you deposit your laboratory protocols in protocols.io to enhance the reproducibility of your results. Protocols.io assigns your protocol its own identifier (DOI) so that it can be cited independently in the future. For instructions see: https://journals.plos.org/plosone/s/submission-guidelines#loc-laboratory-protocols. Additionally, PLOS ONE offers an option for publishing peer-reviewed Lab Protocol articles, which describe protocols hosted on protocols.io. Read more information on sharing protocols at https://plos.org/protocols?utm_medium=editorial-email&utm_source=authorletters&utm_campaign=protocols.

As the corresponding author, your ORCID iD is verified in the submission system and will appear in the published article. PLOS supports the use of ORCID, and we encourage all coauthors to register for an ORCID iD and use it as well. Please encourage your coauthors to verify their ORCID iD within the submission system before final acceptance, as unverified ORCID iDs will not appear in the published article. *Only*  the individual author can complete the verification step; PLOS staff *cannot*  verify ORCID iDs on behalf of authors.

We look forward to receiving your revised manuscript.

Kind regards,

Yoko Hasegawa

Academic Editor

PLOS One

Journal requirements:

When submitting your revision, we need you to address these additional requirements.

1. Please ensure that your manuscript meets PLOS ONE's style requirements, including those for file naming. The PLOS ONE style templates can be found at

https://journals.plos.org/plosone/s/file?id=wjVg/PLOSOne_formatting_sample_main_body.pdf and

https://journals.plos.org/plosone/s/file?id=ba62/PLOSOne_formatting_sample_title_authors_affiliations.pdf

2. The ethical approval number(s) listed in the manuscript and/or submission metadata does not match the approval number on the ethical approval document you provided. Please ensure that all approval numbers are correct.

3. In the online submission form, you indicated that [We open the data if it requested].

All PLOS journals now require all data underlying the findings described in their manuscript to be freely available to other researchers, either 1. In a public repository, 2. Within the manuscript itself, or 3. Uploaded as supplementary information.

This policy applies to all data except where public deposition would breach compliance with the protocol approved by your research ethics board. If your data cannot be made publicly available for ethical or legal reasons (e.g., public availability would compromise patient privacy), please explain your reasons on resubmission and your exemption request will be escalated for approval.

4. Please include captions for your Supporting Information files at the end of your manuscript, and update any in-text citations to match accordingly. Please see our Supporting Information guidelines for more information: http://journals.plos.org/plosone/s/supporting-information.

If the reviewer comments include a recommendation to cite specific previously published works, please review and evaluate these publications to determine whether they are relevant and should be cited. There is no requirement to cite these works unless the editor has indicated otherwise.

Additional Editor Comments:

This is a highly interesting and valuable manuscript. However, revisions are necessary to further improve the quality of the paper.

Please read the reviewers’ comments carefully and revise the manuscript accordingly.

[Note: HTML markup is below. Please do not edit.]

Reviewers' comments:

Reviewer's Responses to Questions

**Comments to the Author**

1. Is the manuscript technically sound, and do the data support the conclusions?

The manuscript must describe a technically sound piece of scientific research with data that supports the conclusions. Experiments must have been conducted rigorously, with appropriate controls, replication, and sample sizes. The conclusions must be drawn appropriately based on the data presented.

Reviewer #1: Partly

Reviewer #2: Yes

Reviewer #3: Partly

**********

2. Has the statistical analysis been performed appropriately and rigorously?

Reviewer #1: Yes

Reviewer #2: Yes

Reviewer #3: Yes

**********

3. Have the authors made all data underlying the findings in their manuscript fully available?

The PLOS Data policy requires authors to make all data underlying the findings described in their manuscript fully available without restriction, with rare exception (please refer to the Data Availability Statement in the manuscript PDF file). The data should be provided as part of the manuscript or its supporting information, or deposited to a public repository. For example, in addition to summary statistics, the data points behind means, medians and variance measures should be available. If there are restrictions on publicly sharing data—e.g. participant privacy or use of data from a third party—those must be specified.

Reviewer #1: No

Reviewer #2: Yes

Reviewer #3: Yes

**********

4. Is the manuscript presented in an intelligible fashion and written in standard English?

PLOS ONE does not copyedit accepted manuscripts, so the language in submitted articles must be clear, correct, and unambiguous. Any typographical or grammatical errors should be corrected at revision, so please note any specific errors here.

Reviewer #1: Yes

Reviewer #2: Yes

Reviewer #3: Yes

**********

5. Review Comments to the Author

Please use the space provided to explain your answers to the questions above. You may also include additional comments for the author, including concerns about dual publication, research ethics, or publication ethics. (Please upload your review as an attachment if it exceeds 20,000 characters)

Reviewer #1: This manuscript addresses an interesting topic and has potential clinical implications. However, several methodological and interpretational issues should be addressed before publication.

① The stated aim of this study is to understand the mechanism of food-related decision-making in the elderly. However, the experimental task only required participants to “look at” or “eat” food, and did not include any actual decision-making process such as choosing between alternative foods. Therefore, interpreting the observed MPFC activity as reflecting food-related decision-making is not fully supported by the experimental design.

② In this study, the control dish (CD) was prepared by the researchers, whereas the preferred dish (PD) was freely selected and purchased by each participant. As a result, the type of food, nutritional composition, taste, appearance, and temperature were not standardized across conditions. Consequently, it is difficult to determine whether the observed differences in MPFC activity reflect food preference or simply differences in the physical and sensory properties of the foods.

③ While the amount of the control dish (CD) was standardized based on caloric intake, the preferred dish (PD) was only required to be “an amount that can be completely consumed,” without controlling for caloric content or portion size. Since satiety and metabolic states such as blood glucose levels can influence brain activity, this lack of control may represent an important confounding factor.

④ Participants were classified as responders when the average z-score exceeded 2. However, the rationale for this threshold is not clearly justified. Given that fNIRS signals are subject to substantial inter-individual variability and noise, the use of a single arbitrary threshold for classification may influence the interpretation of the results.

⑤ Although the total sample size was 68 participants, dividing them into four groups resulted in relatively small sample sizes for each group. Moreover, multiple statistical tests were conducted across numerous questionnaire variables. This raises concerns about the inflation of Type I error due to multiple comparisons.

⑥ The ultimate goal of the study is to understand appetite loss in the elderly; however, the experiments were conducted only in young participants. Since aging is associated with changes in taste, olfaction, cognitive function, and neural activity patterns, caution is required when generalizing the findings from young adults to elderly populations.

⑦ Continuous fNIRS signals were dichotomized into responder and non-responder groups. Such dichotomization may lead to loss of information and reduced statistical power.

⑧ In this study, signals recorded around the Fpz region (cH7–10) were interpreted as MPFC activity. However, due to the relatively low spatial resolution of fNIRS and probe placement, the measured signals may reflect broader prefrontal activity, including the frontopolar cortex (BA10), rather than specific MPFC subregions. In addition, fNIRS cannot adequately measure deeper cortical regions such as the ventromedial prefrontal cortex. Furthermore, eating tasks involve chewing, facial muscle activity, and superficial blood flow changes, which may contaminate the signals. Although motion-related components were removed using a mathematical approach, short-separation channels were not used to remove superficial signals. Therefore, caution is required when interpreting these signals as direct indicators of MPFC activity.

⑨ The study interprets MPFC activity as reflecting food-related decision-making and appetite-related processes. However, the MPFC is known to be involved in multiple functions, including value representation, reward processing, and self-referential processing. In addition, appetite regulation is primarily mediated by subcortical regions such as the hypothalamus. Therefore, attributing the observed MPFC activity specifically to decision-making or appetite regulation may be an overinterpretation, and these findings should be interpreted with caution.

Reviewer #2: Congratulations , I enjoy reading your interested paper.

As you recommended in your paper, I agree with further technical improvements culd be required in order to determine whether the food intake test should be combined with fNIRS to contribute to the treatment of disorders in the elderly with TCA.

Also I completely agree to study younger people around 40 years old.

Reviewer #3: This study used functional near-infrared spectroscopy to investigate the relationship between activity in the food-dependent medial prefrontal cortex (MPFC) and food preferences and consumption frequency; its purpose and methods are quite interesting. However, the explanation of the objectives and methods used to test the research hypothesis appears to be unclear. To improve the quality of the manuscript, I have pointed out the following major and minor issues:

Major　comments

Abstract and Introduction

I would appreciate it if you could clarify the true purpose of this study. The abstract states that　“to clarify the relationship of food dependent medial prefrontal cortex (MPFC) activity with food preference and intake frequency by functional near-infrared spectroscopy (fNIRS) to determine ways to treat the appetite loss and have difficulty in explain what they want to eat.” On the other hand, introduction states that “to clarify the relationship of the activity of MPFC with food intake by fNIRS to find satisfactory methods of mechanism of food eating decision-making in the elderly who have lost their appetite.”

P7L82 The statement mentions “to identify satisfactory methods for understanding the mechanisms underlying food-related decision-making.” Does the research design used in this study—which employs regression analysis to identify food preferences and eating habits that are more likely to vary between responders and non-responders—align with the objective of elucidating decision-making mechanisms? Please confirm whether the research question and the design are consistent.

P7L81 Since the participants in this study were not elderly individuals—but rather young healthy adults selected to establish the methodology—wouldn’t the purpose be “to clarify the relationship between activity in the food-dependent medial prefrontal cortex (MPFC) and food preferences and consumption frequency in young healthy adults, with the aim of establishing methods for assessing food preferences and appetite in elderly individuals with poor appetite”?

P10L132 Although this study aims to establish a methodology using young male and female participants, please explain why the energy intake was set at 34% of the daily energy intake of 1,600 kcal for elderly women. For example, a 544-kcal meal might be too small for young men, potentially leading to an underestimation of their responses.

P9L109 While some studies suggest that dietary preferences and eating behaviors in females fluctuate with their menstrual cycle (ex. PMID: 38342313), were the menstrual cycles of the female participants standardized during the measurement period of this study? If there is no consistency, is it possible to exclude the possibility that periodically fluctuating food preferences influence activity in the medial prefrontal cortex?

Throughout this discussion

This study revealed that MPFC activity when just looking at specific foods or when eating them varies depending on food preferences, meal frequency, and knowledge about food. Please explain the academic or clinical significance of these results in terms of establishing the methods for assessing food preferences and appetite that this study aims to develop.

Minor comments

P15L207 Since ANOVA is an abbreviation, please include (ANOVA) after the full name.

P15L199 When distinguishing between responders and non-responders, a Z-score of more than 2 is often used; however, please clearly state the rationale for this cutoff to ensure readers can easily understand it.

P35 In the CD-responders post titled “Eat bread at breakfast,” there is an extra parenthesis in the SE section, so please correct it.

P39-42 Please check Table 4, as it may be duplicated.

**********

6. PLOS authors have the option to publish the peer review history of their article (what does this mean?). If published, this will include your full peer review and any attached files.

If you choose “no”, your identity will remain anonymous but your review may still be made public.

**Do you want your identity to be public for this peer review?**  For information about this choice, including consent withdrawal, please see our Privacy Policy.

Reviewer #1: No

Reviewer #2: No

Reviewer #3: No

**********

[NOTE: If reviewer comments were submitted as an attachment file, they will be attached to this email and accessible via the submission site. Please log into your account, locate the manuscript record, and check for the action link "View Attachments". If this link does not appear, there are no attachment files.]

To ensure your figures meet our technical requirements, please review our figure guidelines: https://journals.plos.org/plosone/s/figures

You may also use PLOS’s free figure tool, NAAS, to help you prepare publication quality figures: https://journals.plos.org/plosone/s/figures#loc-tools-for-figure-preparation.

NAAS will assess whether your figures meet our technical requirements by comparing each figure against our figure specifications.

# Author comment: Author response to Decision Letter 1

**DOI:** 10.1371/journal.pone.0343481.r002

16 Jun 2026

Reviewer #1: This manuscript addresses an interesting topic and has potential clinical implications. However, several methodological and interpretational issues should be addressed before publication.

① The stated aim of this study is to understand the mechanism of food-related decision-making in the elderly. However, the experimental task only required participants to “look at” or “eat” food, and did not include any actual decision-making process such as choosing between alternative foods. Therefore, interpreting the observed MPFC activity as reflecting food-related decision-making is not fully supported by the experimental design.

As reviewers 1 and 3 pointed out, results of this study not enough to elucidate the decision-making mechanisms involved in looking/eating foods. This is because the decision-making mechanisms are complex, and the spatial resolution of NIRS is not sufficient. As mentioned in below, one of our aims of this study is to clarify the relationship between activity in the food-dependent MPFC and food preferences and consumption frequency in young healthy adults, with the aim of establishing methods for assessing food preferences and appetite in elderly individuals with poor appetite. Thus, we revised the manuscript for focus on this point (Abstract, P3L24-28, Introduction, P7L79-82, L83-87).

② In this study, the control dish (CD) was prepared by the researchers, whereas the preferred dish (PD) was freely selected and purchased by each participant. As a result, the type of food, nutritional composition, taste, appearance, and temperature were not standardized across conditions. Consequently, it is difficult to determine whether the observed differences in MPFC activity reflect food preference or simply differences in the physical and sensory properties of the foods.

The CD amount was selected for feasibility in future studies of older adults, but it may have been insufficient for some young male participants. Changing the concentration of blood sugar may potentially associate with the results of the MFPR activity in this study. This is also one of the limitations of this research, and we plan to address it by improving the methodology in the future. We mentioned it in Discussion (P29L387-L394 and P30L403-410).

③ While the amount of the control dish (CD) was standardized based on caloric intake, the preferred dish (PD) was only required to be “an amount that can be completely consumed,” without controlling for caloric content or portion size. Since satiety and metabolic states such as blood glucose levels can influence brain activity, this lack of control may represent an important confounding factor.

This is also the important point to concern. Blood glucose level can influence the brain activity. To clarified the point, for example, study should be conducted combining continuous blood glucose measurement methods using such as Freestyle Libre (Abbott). To avoid the effect of fullness, we analyzed the data of eating for first ten min in this study. However, this time setting also not established. We plan to modified the methods in future study. We mentioned it in the Discussion (P29L387-L394 and P30L403-410).

④ Participants were classified as responders when the average z-score exceeded 2. However, the rationale for this threshold is not clearly justified. Given that fNIRS signals are subject to substantial inter-individual variability and noise, the use of a single arbitrary threshold for classification may influence the interpretation of the results.

We removed the responder/non-responder classification and analyzed continuous baseline-corrected oxyHb values. We also not made threshold of activity to reduce the Type I error. We mentioned it in Methods (P16L219-P17L230).

⑤ Although the total sample size was 68 participants, dividing them into four groups resulted in relatively small sample sizes for each group. Moreover, multiple statistical tests were conducted across numerous questionnaire variables. This raises concerns about the inflation of Type I error due to multiple comparisons.

Thank you very much for your kind suggestion. We used PCA to reduce dimensionality and report the analyses as exploratory. We hope this modification reduce the concern of reviewer 1.

As shown in revised manuscript, preference of food and gender significantly associated with the amplitude of oxyHb induced by food intake. The knowledge of seasonal foods potentially associated with the preference of foods and thus, we may observe these factors by using fNIRS. Although the methodology need establishing (e.g., what foods appropriate to use as a test food, detecting other factors such as eye movement), we think that fNIRS is useful for detecting some food choice rerating behavior of human (Results of revised manuscript. P18L241-P26L357, Tables 1 to 4).

⑥ The ultimate goal of the study is to understand appetite loss in the elderly; however, the experiments were conducted only in young participants. Since aging is associated with changes in taste, olfaction, cognitive function, and neural activity patterns, caution is required when generalizing the findings from young adults to elderly populations.

As mentioned in discussion, results of this study could not generalize to elderly people because of the change in taste, olfaction, cognitive function and neural activity patterns. However, at least we found the recording of fNIRS during eating food can estimate some food dependent habit and behavior, and thus, modifying the method and observing the response from medial/frontopolar prefrontal region in elderly could be make sense. It is future study. We mentioned it in Discussion (P30L395-P31L410).

⑦ Continuous fNIRS signals were dichotomized into responder and non-responder groups. Such dichotomization may lead to loss of information and reduced statistical power.

As mentioned above, we performed multiple regression analysis directly on response of fNIRS without grouping (Results of revised manuscript. P18L241-P26L357, Tables 1 to 4).

⑧ In this study, signals recorded around the Fpz region (cH7–10) were interpreted as MPFC activity. However, due to the relatively low spatial resolution of fNIRS and probe placement, the measured signals may reflect broader prefrontal activity, including the frontopolar cortex (BA10), rather than specific MPFC subregions. In addition, fNIRS cannot adequately measure deeper cortical regions such as the ventromedial prefrontal cortex. Furthermore, eating tasks involve chewing, facial muscle activity, and superficial blood flow changes, which may contaminate the signals. Although motion-related components were removed using a mathematical approach, short-separation channels were not used to remove superficial signals. Therefore, caution is required when interpreting these signals as direct indicators of MPFC activity.

Thank you very much for your helpful suggestion. We now refer to these as fNIRS signals recorded over the medial/frontopolar prefrontal region. We mentioned it in Methods (P14L194-P15L200) and Discussion (P28L362-372 and P31L413-416).

⑨ The study interprets MPFC activity as reflecting food-related decision-making and appetite-related processes. However, the MPFC is known to be involved in multiple functions, including value representation, reward processing, and self-referential processing. In addition, appetite regulation is primarily mediated by subcortical regions such as the hypothalamus. Therefore, attributing the observed MPFC activity specifically to decision-making or appetite regulation may be an overinterpretation, and these findings should be interpreted with caution.

As mentioned by reviewer 1, we revised the manuscript to avoid interpreting the signals as direct indicators of decision-making or appetite regulation. We hope this revise reduced the concern of reviewer 1. We mentioned it in Discussion (P28L362-372)

Reviewer #2: Congratulations , I enjoy reading your interested paper.

As you recommended in your paper, I agree with further technical improvements culd be required in order to determine whether the food intake test should be combined with fNIRS to contribute to the treatment of disorders in the elderly with TCA.

Also I completely agree to study younger people around 40 years old.

Thank you for your comments. We plan to refine the method and then test it in middle-aged and older adults in future studies.

Reviewer #3: This study used functional near-infrared spectroscopy to investigate the relationship between activity in the food-dependent medial prefrontal cortex (MPFC) and food preferences and consumption frequency; its purpose and methods are quite interesting. However, the explanation of the objectives and methods used to test the research hypothesis appears to be unclear. To improve the quality of the manuscript, I have pointed out the following major and minor issues:

Major　comments

Abstract and Introduction

I would appreciate it if you could clarify the true purpose of this study. The abstract states that　“to clarify the relationship of food dependent medial prefrontal cortex (MPFC) activity with food preference and intake frequency by functional near-infrared spectroscopy (fNIRS) to determine ways to treat the appetite loss and have difficulty in explain what they want to eat.” On the other hand, introduction states that “to clarify the relationship of the activity of MPFC with food intake by fNIRS to find satisfactory methods of mechanism of food eating decision-making in the elderly who have lost their appetite.”

So sorry about our unestablished writing. We revised the Abstract and Introduction to state that this study examined young healthy adults as a methodological and exploratory step toward future studies in older adults. (Abstract, P3L24-28, Introduction, P7L79-82, L83-87).

P7L82 The statement mentions “to identify satisfactory methods for understanding the mechanisms underlying food-related decision-making.” Does the research design used in this study—which employs regression analysis to identify food preferences and eating habits that are more likely to vary between responders and non-responders—align with the objective of elucidating decision-making mechanisms? Please confirm whether the research question and the design are consistent.

What we wanted to do in this study was to examine whether fNIRS could be used as a biosensor for appetite, usual dietary habits, and preferences. For this purpose, initially, we planned to discriminate the responses of fNIRS using a pattern of responder/nonresponder based on z-scores. However, as pointed out by reviewer 1 and you, there was a risk that the selection of the z-score threshold would be unclear. Therefore, in the revised manuscript, we changed the method to look at the regression between the actual oxyHb values and the explanatory variables.

As shown in revised manuscript, preference of food and gender significantly associated with the amplitude of oxyHb induced by food intake. The knowledge of seasonal foods potentially associated with the preference of foods and thus, we may observe these factors by using fNIRS. Although the methodology need establishing (e.g., what foods appropriate to use as a test food, detecting other factors such as eye movement), we think that fNIRS is useful for detecting some food choice rerating behavior of human (Results of revised manuscript. P18L241-P26L357, Tables 1 to 4).

P7L81 Since the participants in this study were not elderly individuals—but rather young healthy adults selected to establish the methodology—wouldn’t the purpose be “to clarify the relationship between activity in the food-dependent medial prefrontal cortex (MPFC) and food preferences and consumption frequency in young healthy adults, with the aim of establishing methods for assessing food preferences and appetite in elderly individuals with poor appetite”?

Thank you very much for your kind suggestion. We mentioned as above, we revised the aim of this study “to clarify the relationship between activity in the food-dependent medial/frontopolar prefrontal regions (MFPR) and food preferences and consumption frequency in young healthy adults, with the aim of establishing methods for assessing food preferences and appetite in elderly individuals with poor appetite using functional near-infrared spectroscopy (fNIRS)” through over (Abstract, P3L24-28, Introduction, P7L79-82, L83-87).

P10L132 Although this study aims to establish a methodology using young male and female participants, please explain why the energy intake was set at 34% of the daily energy intake of 1,600 kcal for elderly women. For example, a 544-kcal meal might be too small for young men, potentially leading to an underestimation of their responses.

In this study, as in the conditions of PD, the aim is to conduct the experiment with an amount that can be fully consumed. To make it applicable to the elderly in the future, we set the amount of CD even older women can finish eating. As you pointed out, for example, young men may have reactions due to it being insufficient. For that reason, we analyzed it at 10 minutes after the start of eating to exclude response changes due to fullness. Issues regarding the amount of CD are planned to be addressed as improvements in methodology in the future. We mentioned it in Discussion (P29L387-P30L394)

P9L109 While some studies suggest that dietary preferences and eating behaviors in females fluctuate with their menstrual cycle (ex. PMID: 38342313), were the menstrual cycles of the female participants standardized during the measurement period of this study? If there is no consistency, is it possible to exclude the possibility that periodically fluctuating food preferences influence activity in the medial prefrontal cortex?

As mentioned by reviewer 3, menstrual cycles certainly affect the results of our study. As reported previously, activity of MPFC induced by odor shows gender and age dependency (Takatsuru et al., 2022). Because menstrual cycle phase was not recorded, we cannot exclude its possible influence and have added this as a limitation. We have added this as a limitation in Discussion (P29L380-386).

Throughout this discussion

This study revealed that MPFC activity when just looking at specific foods or when eating them varies depending on food preferences, meal frequency, and knowledge about food. Please explain the academic or clinical significance of these results in terms of establishing the methods for assessing food preferences and appetite that this study aims to develop.

Although NIRS has insufficient spatial resolution and its signals are a mixture of multiple brain functions, observing MPFC activity during eating using fNIRS may allow for estimation of food preferences and intake frequency in participants (may including the elderly). Using fNIRS as a biosensor in combination with food intake tests may contribute to the treatment of food-related disorders (such as anorexia nervosa) in many age groups, including the elderly. However, further technological improvements will be needed to determine whether it has definitive clinical significance. We mentioned it in Conclusion of Discussion (P31L413-419)

Minor comments

P15L207 Since ANOVA is an abbreviation, please include (ANOVA) after the full name.

Thank you for your suggestion. We revised the part (P15L212-214).

P15L199 When distinguishing between responders and non-responders, a Z-score of more than 2 is often used; however, please clearly state the rationale for this cutoff to ensure readers can easily understand it.

As mentioned above, threshold of z-score was depended on the change in z-score during baseline recording. Almost all z-score during baseline was ±2. It is well known that fNIRS responses contain a lot of individual differences and noise, and based on the purpose of this study (to clarify whether fNIRS can be used as a biosensor to estimate subjects' food preferences and eating behavior), we deliberately analyzed the measurements oxyHb after simple baseline correction, with the intention of providing a foundation for future research (Methods of revised manuscript, P14L191-194).

P35 In the CD-responders post titled “Eat bread at breakfast,” there is an extra parenthesis in the

Attachment Submitted filename: Reviewer.docx

# Peer review: Decision Letter 1

**DOI:** 10.1371/journal.pone.0343481.r003

**Authors:** Yoko Hasegawa

30 Jun 2026

Food preference and gender are associated with medial/frontopolar prefrontal regions functional near-infrared spectroscopy responses during eating: an exploratory study in young adults.

PONE-D-26-06160R1

Dear Dr. Takatsuru,

We’re pleased to inform you that your manuscript has been judged scientifically suitable for publication and will be formally accepted for publication once it meets all outstanding technical requirements.

Within one week, you’ll receive an e-mail detailing the required amendments. When these have been addressed, you’ll receive a formal acceptance letter and your manuscript will be scheduled for publication.

An invoice will be generated when your article is formally accepted. Please note, if your institution has a publishing partnership with PLOS and your article meets the relevant criteria, all or part of your publication costs will be covered. Please make sure your user information is up-to-date by logging into Editorial Manager at Editorial Manager® and clicking the ‘Update My Information' link at the top of the page. For questions related to billing, please contact billing support.

If your institution or institutions have a press office, please notify them about your upcoming paper to help maximize its impact. If they’ll be preparing press materials, please inform our press team as soon as possible -- no later than 48 hours after receiving the formal acceptance. Your manuscript will remain under strict press embargo until 2 pm Eastern Time on the date of publication. For more information, please contact onepress@plos.org.

Kind regards,

Yoko Hasegawa

Academic Editor

PLOS One

Additional Editor Comments (optional):

Reviewers' comments:

Reviewer's Responses to Questions

**Comments to the Author**

1. If the authors have adequately addressed your comments raised in a previous round of review and you feel that this manuscript is now acceptable for publication, you may indicate that here to bypass the “Comments to the Author” section, enter your conflict of interest statement in the “Confidential to Editor” section, and submit your "Accept" recommendation.

Reviewer #1: All comments have been addressed

Reviewer #2: (No Response)

**********

2. Is the manuscript technically sound, and do the data support the conclusions?

The manuscript must describe a technically sound piece of scientific research with data that supports the conclusions. Experiments must have been conducted rigorously, with appropriate controls, replication, and sample sizes. The conclusions must be drawn appropriately based on the data presented.

Reviewer #1: Yes

Reviewer #2: Yes

**********

3. Has the statistical analysis been performed appropriately and rigorously?

Reviewer #1: Yes

Reviewer #2: Yes

**********

4. Have the authors made all data underlying the findings in their manuscript fully available?

The PLOS Data policy requires authors to make all data underlying the findings described in their manuscript fully available without restriction, with rare exception (please refer to the Data Availability Statement in the manuscript PDF file). The data should be provided as part of the manuscript or its supporting information, or deposited to a public repository. For example, in addition to summary statistics, the data points behind means, medians and variance measures should be available. If there are restrictions on publicly sharing data—e.g. participant privacy or use of data from a third party—those must be specified.

Reviewer #1: Yes

Reviewer #2: Yes

**********

5. Is the manuscript presented in an intelligible fashion and written in standard English?

PLOS ONE does not copyedit accepted manuscripts, so the language in submitted articles must be clear, correct, and unambiguous. Any typographical or grammatical errors should be corrected at revision, so please note any specific errors here.

Reviewer #1: Yes

Reviewer #2: Yes

**********

6. Review Comments to the Author

Please use the space provided to explain your answers to the questions above. You may also include additional comments for the author, including concerns about dual publication, research ethics, or publication ethics. (Please upload your review as an attachment if it exceeds 20,000 characters)

Reviewer #1: I appreciate the authors' careful revisions and thoughtful responses to my previous comments. The manuscript has been substantially improved, and my major concerns have been adequately addressed.

In particular, the clarification of the study aims, the replacement of the responder/non-responder classification with continuous fNIRS analyses, the more appropriate interpretation of the prefrontal fNIRS signals, and the more balanced discussion have considerably strengthened the manuscript.

I have no further major or minor comments. I believe the manuscript is now suitable for publication in PLOS ONE.

Reviewer #2: (No Response)

**********

7. PLOS authors have the option to publish the peer review history of their article (what does this mean?). If published, this will include your full peer review and any attached files.

If you choose “no”, your identity will remain anonymous but your review may still be made public.

**Do you want your identity to be public for this peer review?**  For information about this choice, including consent withdrawal, please see our Privacy Policy.

Reviewer #1: No

Reviewer #2: No

**********

# Editor report: Acceptance letter

**DOI:** 10.1371/journal.pone.0343481.r004

**Authors:** Yoko Hasegawa

PONE-D-26-06160R1

PLOS One

Dear Dr. Takatsuru,

I'm pleased to inform you that your manuscript has been deemed suitable for publication in PLOS One. Congratulations! Your manuscript is now being handed over to our production team.

At this stage, our production department will prepare your paper for publication. This includes ensuring the following:

* All references, tables, and figures are properly cited

* All relevant supporting information is included in the manuscript submission,

* There are no issues that prevent the paper from being properly typeset

You will receive further instructions from the production team, including instructions on how to review your proof when it is ready. Please keep in mind that we are working through a large volume of accepted articles, so please give us a few days to review your paper and let you know the next and final steps.

Lastly, if your institution or institutions have a press office, please let them know about your upcoming paper now to help maximize its impact. If they'll be preparing press materials, please inform our press team within the next 48 hours. Your manuscript will remain under strict press embargo until 2 pm Eastern Time on the date of publication. For more information, please contact onepress@plos.org.

You will receive an invoice from PLOS for your publication fee after your manuscript has reached the completed accept phase. If you receive an email requesting payment before acceptance or for any other service, this may be a phishing scheme. Learn how to identify phishing emails and protect your accounts at https://explore.plos.org/phishing.

If we can help with anything else, please email us at customercare@plos.org.

Thank you for submitting your work to PLOS ONE and supporting open access.

Kind regards,

PLOS ONE Editorial Office Staff

on behalf of

Dr. Yoko Hasegawa

Academic Editor

PLOS One
